# A Dual-Modality CNN Approach for RSS-Based Indoor Positioning Using Spatial and Frequency Fingerprints

**DOI:** 10.3390/s25175408

**Published:** 2025-09-02

**Authors:** Xiangchen Lai, Yunzhi Luo, Yong Jia

**Affiliations:** 1 School of Mechanical and Electrical Engineering, Chengdu University of Technology, Chengdu 610059, China; 202221020612@stu.cdut.edu.cn; 2 School of Chemical Engineering and Technology, Xi’an Jiaotong University, Xi’an 710049, China; xixixixi_198@163.com

**Keywords:** fingerprinting indoor positioning, radio signal strength, convolutional neural network, cross-modality

## Abstract

Indoor positioning systems based on received signal strength (RSS) achieve indoor positioning by leveraging the position-related features inherent in spatial RSS fingerprint images. Their positioning accuracy and robustness are directly influenced by the quality of fingerprint features. However, the inherent spatial low-resolution characteristic of spatial RSS fingerprint images makes it challenging to effectively extract subtle fingerprint features. To address this issue, this paper proposes an RSS-based indoor positioning method that combines enhanced spatial frequency fingerprint representation with fusion learning. First, bicubic interpolation is applied to improve image resolution and reveal finer spatial details. Then, a 2D fast Fourier transform (2D FFT) converts the enhanced spatial images into frequency domain representations to supplement spectral features. These spatial and frequency fingerprints are used as dual-modality inputs for a parallel convolutional neural network (CNN) model with efficient multi-scale attention (EMA) modules. The model extracts modality-specific features and fuses them to generate enriched representations. Each modality—spatial, frequency, and fused—is passed through a dedicated fully connected network to predict 3D coordinates. A coordinate optimization strategy is introduced to select the two most reliable outputs for each axis (x, y, z), and their average is used as the final estimate. Experiments on seven public datasets show that the proposed method significantly improves positioning accuracy, reducing the mean positioning error by up to 47.1% and root mean square error (RMSE) by up to 54.4% compared with traditional and advanced time–frequency methods.

## 1. Introduction

Indoor positioning systems have demonstrated significant value in emerging applications such as location-based services (LBSs), Internet of Things (IoT), and 5G networks [1,2,3]. Traditional outdoor positioning technology, such as the Global Positioning System (GPS), fails to meet high-precision positioning requirements in indoor environments due to building obstructions and multipath effects [4]. As a result, various wireless technology-based indoor positioning solutions, such as WiFi, Bluetooth, and ultra-wideband (UWB), have gradually developed [5].

Among wireless signal-based technologies, WiFi and Bluetooth fingerprint hold a prominent position in indoor positioning scenarios as two standardized technologies that predominantly utilize received signal strength (RSS) or channel state information (CSI) for location estimation. Compared with RSS, CSI provides richer signal characterization data, thereby offering greater stability and accuracy [6]. However, CSI implementation requires advanced network interface cards (NICs) that are not yet ubiquitously integrated into smartphone Bluetooth/WiFi modules [7]. In contrast, most wireless NIC-enabled systems inherently provide RSS measurements, and RSS values are widely and freely accessible across mobile devices [8]. RSS-based indoor positioning technologies are primarily categorized into geometric and fingerprint-based methodologies. Geometric approaches estimate location using parameters such as time of flight (ToF) and angle of arrival (AoA) [9], yet demonstrate limited effectiveness in complex indoor environments. The fingerprinting localization method constructs a fingerprint database by pre-collecting signal characteristics from different access points (APs) at various reference points (RPs) and determines the optimal position through matching models. Without requiring prior knowledge of base station locations, time, or angle measurements, this approach achieves low-cost and high-efficiency positioning, demonstrating promising application prospects. Our study focuses on the RSS fingerprinting indoor positioning technology.

Traditional machine learning (ML)-based pattern matching methods, including K-nearest neighbor (KNN) algorithm [10] and support vector machine (SVM) [11], have achieved robust positioning but struggle to learn features in complex dynamic environments with high-dimensional RSS sequences, limiting their potential for high precision. In contrast, neural network methods enhance feature extraction capabilities for high-dimensional RSS sequences, improving positioning accuracy and robustness. For instance, in [12], the deep neural network (DNN), an efficient neural network that automatically abstracted RSS sequence features through its multi-layer architecture, was introduced and demonstrated superior positioning accuracy compared with traditional machine learning methods. In [13], DNN has been further improved by adding a differentiated thresholded rectified linear unit, which filtered low probability coordinates to improve the accuracy and robustness of the 3D positioning task. In [14], the CNNLoc framework was proposed, which integrated stacked autoencoders (SAEs) with a 1D CNN to extract critical features from high-dimensional RSS sequences and realized a high-accuracy floor positioning. Introducing neural networks has brought better accuracy and robustness, but simple 1D neural networks may not be sufficient for handling high-dimensional RSS data and complex positioning scenarios. To further utilize neural networks and better process high-dimensional RSS data, many studies have converted RSS sequences into 2D fingerprint images and combined them with CNN models. In [15,16,17,18,19,20], the 2D CNN-based system was widely utilized, which constructed convolutional network layers with fully connected layers to realize the classification or regression positioning task by giving the RSS-generated 2D images as inputs. Moreover, ref. [16] combined extreme learning machine autoencoder (ELM-AE) with a 2D CNN structure with max pooling layers and fully connected layers, realizing effective 2D positioning and floor classification. Ref. [20] proposed an innovative CNN-based model, which introduced the efficient channel attention (ECA) module behind each convolutional layer and significantly enhanced the positioning accuracy. The 2D fingerprint image format can effectively handle high-dimensional RSS data, and the superiority of CNN in image tasks can also be well utilized or further enhanced, providing a new practical method for boosting fingerprinting indoor positioning.

Cross-modality methods have been integrated into fingerprinting indoor positioning to enhance positioning performance further by incorporating the data of different modalities [21,22]. Ref. [23] combined WiFi RSS signals with several UWB beacons for positioning and achieved sub-meter-level positioning accuracy. Ref. [19] proposed a positioning method that converts Bluetooth RSS signals into fingerprint images to train the CNN for classifying floors and locating areas and to combine magnetic field data matching to determine unknown point coordinates, effectively solving the problems of floor judgment and large-scale application in traditional fingerprint positioning. Ref. [20] combined visible light 2D fingerprints and Bluetooth 2D fingerprints to generate a hybrid 2D fingerprint for more accurate positioning. The combination of multi-modal data can effectively improve positioning performance. However, these works all focused on data obtained from different types and devices and neglected more accessible multi-modality in one data form’s different domain.

Time–frequency technology has recently brought a new approach to improving positioning accuracy and robustness in recent years. Ref. [24] proposed a filtering method based on the Fourier transform to obtain better positioning performance by preprocessing RSS sequence values in the frequency domain. Ref. [25] proposed a filtering method based on wavelet scattering transform to preprocess RSS sequence values in the frequency domain. Furthermore, ref. [26] proposed a joint time–frequency RSS method, using the continuous wavelet transform to extract the joint time–frequency spectrogram of each raw RSS data and use this image fingerprint with a CNN to position. Data processing through time–frequency techniques can easily achieve better positioning performance without additional hardware support or complex network structures.

Motivated by these works, we first introduce a cross-modality model based on RSS fingerprint images’ spatial and frequency domains and combine the outputs of different modalities for final positioning, achieving better accuracy and robustness. Moreover, to the best of our knowledge, we first utilize bicubic interpolation for the data enhancement of RSS 2D fingerprint images for a better feature study. The main contributions of this paper are as follows:

- The RSS sequence of each RP is converted into 2D spatial fingerprint images together with its frequency domain fingerprint images through the 2D FFT, thus creating two modalities’ fingerprint information of each RP.
- Bicubic interpolation is introduced to reconstruct higher-resolution fingerprint images with more detailed features through 2× super-resolution.
- An innovative cross-modality deep learning model for 3D indoor positioning is proposed. Our model utilizes spatial fingerprint and frequency domain fingerprint for cross-modality fusion and joint prediction and combines the advanced efficient multi-scale attention (EMA) module.
- Experimentation evaluations are performed in seven publicly available datasets. The results validate that our cross-modality deep learning method significantly enhances positioning accuracy and robustness.

## 2. Methods

**Figure 1:**
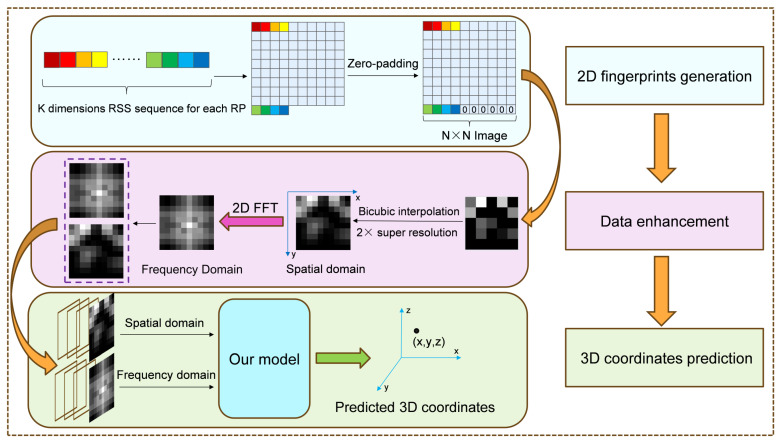
Overview of the proposed indoor positioning method.

The simplified overview of our proposed fingerprinting indoor positioning method is shown in Figure 1. In the 2D fingerprint images generation stage, the RSS sequence of each RP is converted into a 2D grayscale fingerprint image. In the data augmentation stage, the bicubic interpolation is introduced to enhance the features of all fingerprint images. At the same time, 2D FFT is performed on the fingerprint image after bicubic interpolation to obtain its frequency domain fingerprint images. In the coordinate prediction stage, the spatial domain fingerprint and the frequency domain fingerprint of each RP are fed into our proposed cross-modality model to give out the predicted 3D coordinates.

### 2.1. Data Preprocessing

Outliers are first excluded from the datasets by setting their values to zero. For the remaining valid RSS values, minimum-based normalization is performed to transform the data into a positive numerical range, which can be expressed as:

(1) $$f\left(x\right)=\frac{x-\min(x_{\mathrm{valid}})}{\max\left(x_{\mathrm{valid}}\right)-\min\left(x_{\mathrm{valid}}\right)}$$

After normalization, a quadratic power transformation is applied to each RSS value to better improve neural network learning. The RSS value sequences for each RP are then scaled to the grayscale pixel range (0–255) by multiplying by 255. Subsequently, the RSS sequences in each RP are reconstructed as N×N grayscale maps, where missing values are automatically padded with zeros.

### 2.2. Bicubic Interpolation

The resolution of RSS fingerprint images depends on the fixed number of APs, and the final prediction accuracy is largely influenced by the fingerprint images in fingerprinting positioning methods. Fingerprint images with more detailed information tend to bring better model learning and positioning results. Therefore, super-resolution methods are considered to enlarge the details of fingerprint images, thereby enhancing the feature expression.

The commonly used super-resolution methods are primarily divided into traditional bilinear interpolation, bicubic interpolation, and deep learning-based super-resolution methods [27,28,29]. The super-resolution method based on deep learning requires the introduction of additional models for additional training, which cannot be well integrated with the positioning model in real time. Therefore, we mainly consider traditional bilinear interpolation and bicubic interpolation methods. Bilinear interpolation and bicubic interpolation are both interpolated by weighting neighboring points, which is simple to operate and has good real-time performance. The difference between the two methods is the number of reference neighboring points. Bilinear interpolation only refers to the pixel values of 4 adjacent points for interpolation, while bicubic interpolation refers to the pixel values of 16 adjacent points for interpolation, which has a much better degree of detail restoration than bilinear interpolation [28]. Therefore, we adopt bicubic interpolation as the method for the super-resolution of fingerprint images. Specifically, bicubic interpolation uses 16 points in the 4 × 4 domain near the interpolation point for interpolation. The function W(x) in Formula (2) is used to allocate weights for 16 reference points and a=−0.5. The super-resolution process can be expressed as Formula (3). Moreover, the size of the fingerprint images constructed from the dataset used in this paper is clearly shown in Table 1.

(2) $$W\left(x\right)=\left\{\begin{array}{cc} {\left(a+2\right)|x|}^{3}-\left(a+3\right){\left|x\right|}^{2}+1, & |x|\leq \;1\\ {a|x|}^{3}-{5a|x|}^{2}+8a\left|x\right|-4a, & 1<|x|\leq \;2\\ 0, & \mathrm{else} \end{array}\right.$$

(3) $$I\left(x,y\right)=\sum_{i=0}^{3}\sum_{j=0}^{3}I\left(x_{i},y_{j}\right)W\left(x-x_{i}\right)W\left(y-y_{j}\right)$$

**Figure 2:**
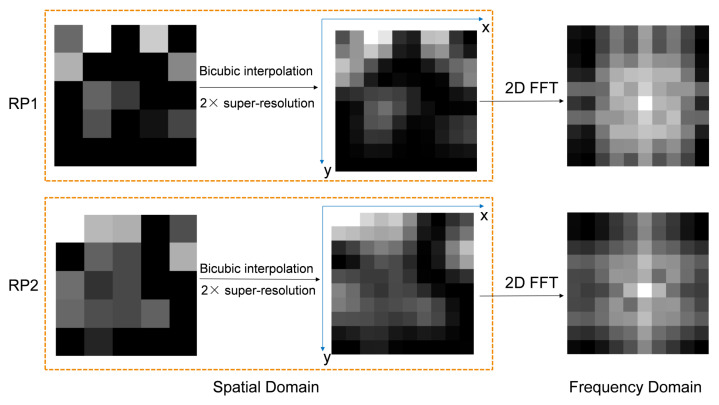
Overview of the bicubic interpolation and 2D FFT processes.

After bicubic interpolation, 2D FFT is used to obtain the frequency domain fingerprint image of each super-resolution image. Frequency domain images have more details than spatial domain images, and even small changes in the spatial domain are magnified in the frequency domain. This characteristic can obviously be utilized to achieve more accurate indoor positioning. The visualization of the entire process is shown in Figure 2. Bicubic interpolation effectively adds a lot of details while preserving the main features of the image, and at the same time, frequency domain images bring more unique information than spatial domain images.

### 2.3. Deep Learning Model

#### 2.3.1. EMA Module

The differences in fingerprint images often occur in very detailed areas, which places high demands on neural networks for learning image details. The attention mechanism module is an available method that improves performance by applying attention to the input feature images to make the network more focused on learning their effective parts.

**Figure 3:**
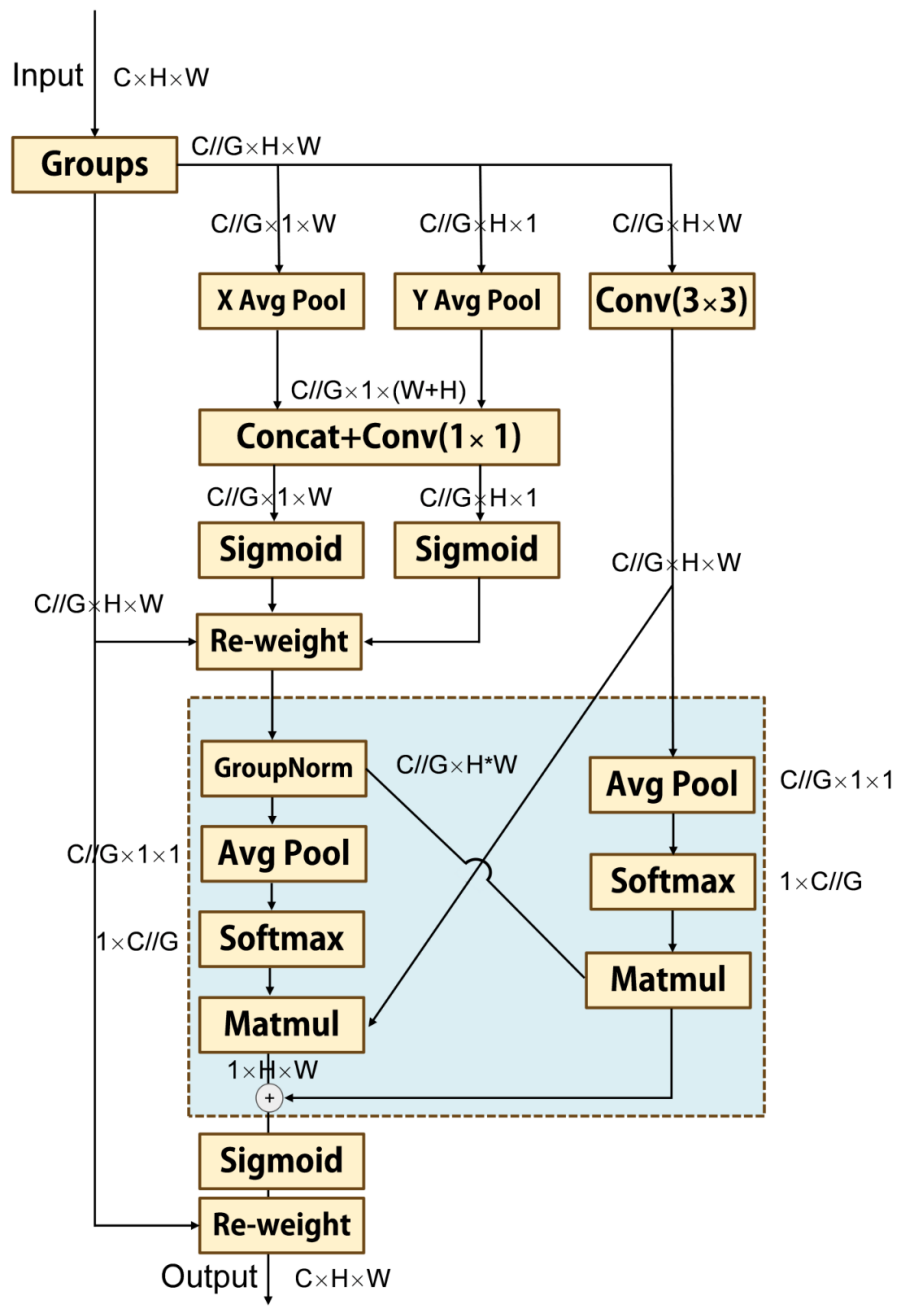
Structure diagram of the EMA module.

Currently, there are many standard attention mechanism modules. Efficient multi-scale attention (EMA) [34] is a type of attention mechanism module. EMA can bring higher performance to neural networks without significantly improving the number of parameters. The structural diagram of EMA is shown in Figure 3. EMA adopts a grouping structure and cross-spatial learning method to capture the dependencies and multi-scale features of short and long distances through a multi-scale dual branch parallel network. Specifically, EMA uses 1 × 1 and 3 × 3 parallel convolutional branches to capture and fuse multi-scale features across dimensions, achieving cross-spatial learning. The spatial semantic features are evenly distributed in each feature map, and short-term and long-term dependencies are effectively established through feature grouping and the multi-scale structure of EMA. Due to the integration of contextual information at different scales, EMA brings more precise pixel-level attention to neural networks on feature maps. Meanwhile, due to the efficient dual parallel branch structure and grouping processing method of EMA, it does not significantly increase the parameter count of neural networks. By introducing EMA into our proposed cross-modality model, our model can better learn the features in fingerprint images, thereby achieving more accurate and robust positioning.

#### 2.3.2. Proposed Cross-Modality Model

**Figure 4:**
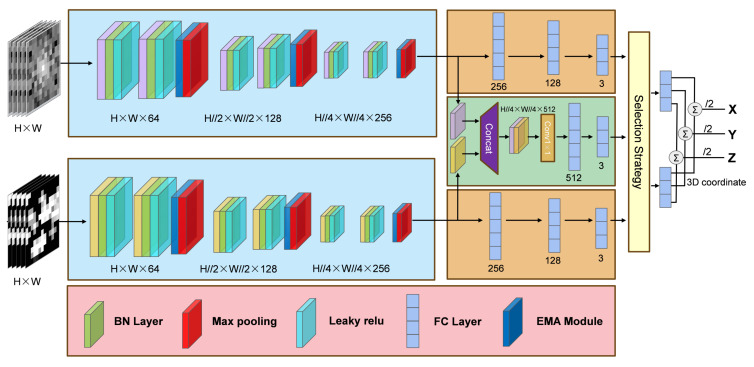
Overall network architecture of our proposed cross-modality model.

The overall framework of our proposed cross-modality model is shown in Figure 4. Our proposed cross-modality model is mainly divided into two parts: the feature extraction part for different input modalities and the multi-modality multi-prediction head part.

The feature extraction part is primarily divided into convolutional layers and EMA modules. We have adopted the design concept of the very deep convolution (VGG) network [35] for the convolutional layer, using non-dimensionality reducing convolution and pooling layers to extract fingerprint image features better. The practicality of the VGG network structure in indoor positioning is verified in [36,37]. Differently, we only design six layers of 3 × 3 non-dimensionality reduction convolution with channels of 64, 128, 256 neurons and three layers of 2 × 2 max pooling behind each two convolution layers to better adapt to the feature learning of relatively low-resolution fingerprint images. An EMA module is introduced after the convolutional layer, which brings an attention mechanism to help the model learn the valid features in the fingerprint image more effectively.

The prediction head part uses multiple fully connected layers to output the 3D coordinates directly. We design a multi-modality (frequency domain, spatial domain, fusion) parallel structure for the prediction head. The frequency domain and spatial domain prediction take the feature maps after feature extraction as inputs and directly output 3D coordinates through fully connected layers of 256, 128, and 64 neurons. For the cross-modality prediction head, the feature maps extracted from the two modalities are first concatenated into a multi-channel feature map with 512 channels. The fused feature maps are then enhanced using a non-dimensionality reduction 1 × 1 convolution kernel to improve the fusion effect. Finally, the fused feature map outputs the 3D coordinates directly through the fully connected layers of 512 and 3 neurons. Through parallel three-branch prediction heads, we simultaneously obtained the 3D coordinates predicted by the frequency domain branch, the spatial domain branch, and the fusion branch. At this time, we design a selection strategy that is supposed to select the two most reliable coordinates from three different dimensions (x/y/z) obtained from different branches, calculates the mean, and then obtains the final 3D coordinate output. Specifically, on the three different coordinate dimensions of x, y, and z, we first determine the median of the three branch data, then based on the absolute value of the difference between the remaining two branch data and the median, leave the data with the smaller absolute value and average it with the median data. Then, we concatenate the coordinates obtained in each dimension to output the final predicted 3D coordinate. This selection strategy aims to utilize the prediction results of different modalities to eliminate large errors while achieving more accurate positioning.

In addition, a batch normalization (BN) layer is added after each convolutional layer to improve the robustness and effectiveness of the network training. Meanwhile, we employ the leaky relu activation function for each hidden layer. Leaky relu introduces a small linear component for negative input, which alleviates the problem of partial neuron deactivation in relu, effectively improves model robustness, and accelerates model convergence [38]. The function expression of leaky relu is as follows:

(4) $$f\left(x\right)=\max\left(ax,x\right)=\left\{\begin{array}{cc} 0.01x, & \mathrm{if}\;x<0\\ x, & \mathrm{if}\;x\geq 0 \end{array}\right.$$

For the selection of the loss function, our proposed model achieves the regression positioning task through fully connected layers. Therefore, we choose MSE as the loss function for the three prediction branches. In the formula, q is the true 3D coordinate label and p is the predicted 3D coordinate label.

(5) $$L_{\mathrm{MSE}}\left(p,q\right)=\frac{1}{n}\sum_{i=1}^{n}{\left(q_{i}-p_{i}\right)}^{2}$$

In the end, the overall loss function of our proposed model is:

(6) $$L=L_{frequency}+L_{spatial}+L_{fusion}.$$

## 3. Experiment Setup

### 3.1. Datasets and Evaluation Metrics

We set our experiments on 2 IEEE 802.11 WiFi and 5 Bluetooth low-energy (BLE) RSS-based fingerprinting indoor positioning datasets, and all datasets are built on real-world measurements. All considered datasets are available online [30,31,32,33], and their practicality has been proven by the prior literature [13,39]. All datasets are unambiguously divided into training and testing subsets and contain APs’ RSS sequences as features together with x, y, z coordinates as labels q=[xyz] for each sample. These datasets vary in data density, technology, environment, number of RPs and APs, and many other dimensions, so our models are evaluated on heterogeneous data. The specific number of samples is shown in Table 2. Table 2 also includes the number of AP numbers, area ranges, and techniques used for each dataset.

We take the 3D positioning error as our main evaluated metric, which is computed as the Euclidean distance between the estimated 3D coordinate label p and the real 3D coordinate label q, expressed as:

(7) $$E_{3D}\left(p,q\right)=\sqrt{{\left(q_{i}-p_{i}\right)}^{2}}$$

Additionally, we take the root mean square error (RMSE) into consideration to clearly show the robustness of each model. The RMSE is computed as:

(8) $$\mathrm{RMSE}=\sqrt{\frac{1}{N}\sum_{i=1}^{N}{\left(q_{i}-p_{i}\right)}^{2}}$$

### 3.2. Considered Benchmark Solutions

In this section, we briefly describe our benchmarks: the CNN-based solution, ANN-based solution, commonly utilized KNN models, and another advanced frequency–time solution. To further validate the improvement effect of our proposed model, we take one branch from our proposed cross-modality model and removed its BN layer and EMA module while using relu activation. Specifically, our CNN benchmark consists of six layers of 3 × 3 non-dimensionality reduction convolution with channels of 64, 128, and 256 neurons and three layers of 2 × 2 max pooling behind each of the two convolution layers, and it finally uses fully connected layers with 256 and 128 neurons and 3 neurons to give the 3D coordinates. We also introduce the DNN benchmark set in paper [13,40,41] as our ANN benchmark. The DNN model consists of an input layer determined by the length of the feature vector after zero padding and three fully connected layers with 128 neurons. Each neuron is activated by relu, and the final output of the fully connected layer has three neurons, directly outputting 3D positioning coordinates. The DNN and CNN benchmark consider MSE loss. Moreover, in the upcoming evaluations and assessments, we consider additional KNN-based benchmarks that are widely recognized as solutions for indoor positioning. According to papers [13,26,39], we have implemented a simple yet effective nonparametric model KNN with K = 1 and the L1 similarity metric (Manhattan distance), which we refer to as the 1NN benchmark. As the improved version of the algorithm interpolates between the neighbors based on their similarity distance, a weighted KNN with K = 3 is also implemented, denoted as the W3NN benchmark. Moreover, we further compare our proposed cross-modality model with another advanced time–frequency positioning method set in paper [26], which uses a feature extraction method based on continuous wavelet transform to convert one-dimensional RSS data into a 2D time–frequency fingerprint image and combines it with CNN to output the final coordinates.

### 3.3. Implementation Details

The numerical evaluation and implementation of the experiments were carried out using a Python 3.8 environment utilizing Scipy, Pandas, Math, Numpy, Scikit-learn, Cv2, Torch==1.7.1, Torchvision==0.7.2, and Torchaudio==0.8.2. We divided the training and validation sets into an 8:2 ratio to prevent overfitting during training. Additionally, we introduced an early stopping mechanism (patience value = 10) to obtain the best model performance promptly and introduced a dropout layer (probability = 0.3) to prevent overfitting better. We used the Adam optimizer for up to 600 rounds during training, with a batch size of 16.

The preprocessed data, consisting of processed 2D fingerprint images (spatial and frequency domains), served as input data for our proposed cross-modality model. The spatial domain fingerprint images were input data for other models.

## 4. Results and Discussion

### 4.1. Models Performance

**Figure 5:**
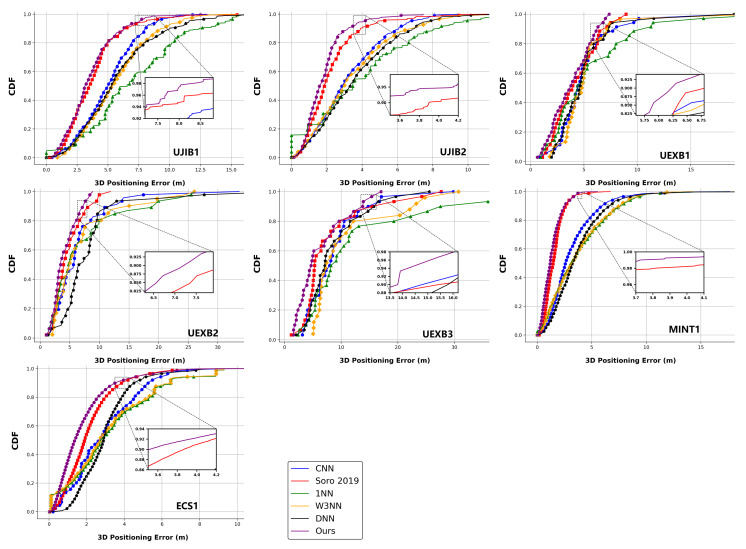
Individual CDFs of the positioning errors with different models on all datasets.

Below, we present the experimental results of all models on all seven datasets. Table 3 shows the mean 3D positioning error results, Table 4 shows the RMSE results, and Figure 5 exhibits the cumulative distribution function (CDF) curves. The experimental results show that our proposed cross-modality model performs best in all seven datasets, with an average positioning error improvement of 0.2–2.8 m. Compared with the advanced time–frequency method in [26], there is still a maximum improvement of 0.5 m. Under such positioning accuracy, our proposed cross-modality model significantly reduces RMSE from 3.9 m to 0.5 m. These can also be clearly seen in CDF curves. In the CDF curves, our proposed cross-modality model always converges to one at the fastest speed to avoid larger errors. The above sufficient experimental results have verified our proposed cross-modality model’s improvement in positioning accuracy while reducing RMSE to enhance the robustness of positioning.

**Figure 6:**
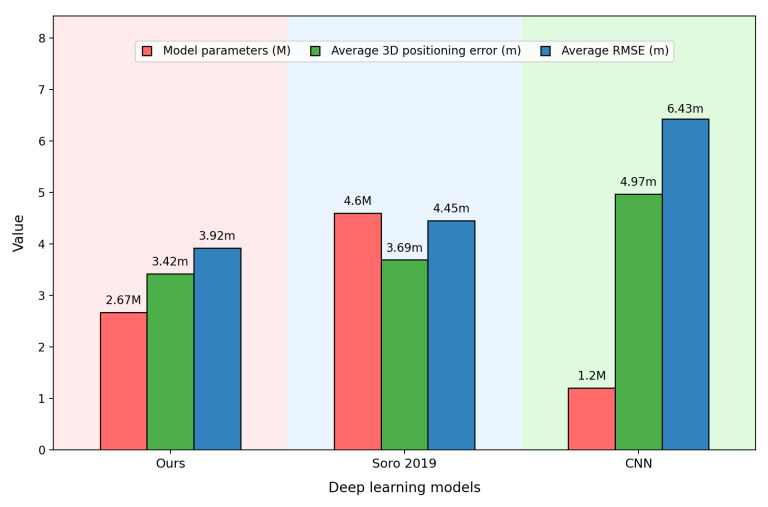
Each deep learning models’ parameters and average positioning error with RMSE.

Furthermore, to validate the effectiveness of our proposed model, statistical analyses are conducted on the parameter counts of all deep learning models. Figure 6 presents the parameter counts, average positioning errors, and RMSEs of different deep learning models. Among them, the CNN model, serving as a simple basic model, has the smallest parameter count (only 1.2 M), but it also brings the largest positioning error and RMSE. Our proposed model, built upon the CNN model, achieves significant improvements in positioning accuracy and RMSE through the integration of dual parallel CNN branches and the EMA module, supplemented by fusion learning and a multi-modal output joint prediction strategy. Compared with the CNN model, our proposed model enhances positioning accuracy by 30% and RMSE by 40%, with only an additional 1.5 M parameters introduced. In comparison with the selected contrastive time–frequency method, our proposed model still has a 42% lower parameter count, while both positioning accuracy and RMSE are improved by approximately 10%. The above results fully demonstrate that the proposed model can effectively enhance positioning performance without significantly increasing parameters or even with a reduction in parameters.

### 4.2. Modalities Performance

**Figure 7:**
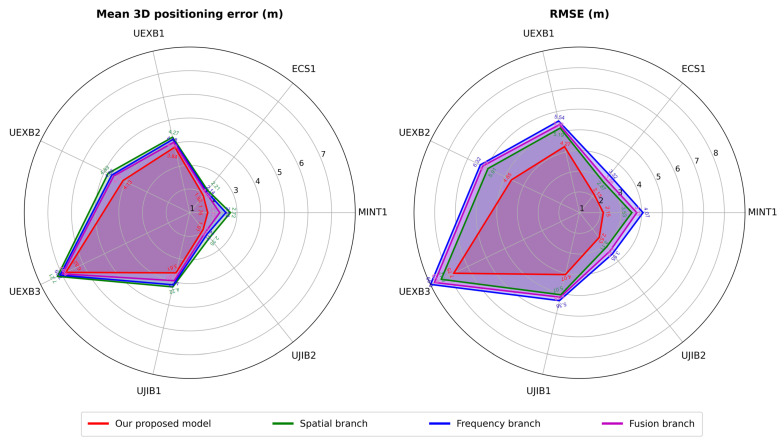
Performance evaluation results of mean 3D positioning error and RMSE on different modalities of our proposed cross-modality on all datasets.

We conducted comparative experiments on multi-modal data to further validate the effectiveness of the proposed cross-modality model. Because the final output of our cross-modality model is an average of two of the modality detection heads, we separately present the output data of the three detection heads for comparison. Figure 7 shows the different modalities’ and our proposed cross-modality model’s mean 3D positioning error and RMSE on all seven datasets. Through experimental data, we find that the average positioning error from the frequency branch is smaller than that of the spatial branch, reducing from 0.2 m to 0.05 m. At the same time, the RMSE of the frequency branch is much larger than that of the spatial branch, with an average increase of about 0.5 m. This reduction in accuracy yet increase in RMSE may indicate that more details in frequency domain fingerprints may bring better performance to neural network positioning models, while these abundant details could also lead to significant errors. The fusion branch brings improvements with 0.1 m accuracy and about 0.3 m reduction in RMSE. Finally, our proposed cross-modality model realizes substantial enhancements. The selection strategy utilizes the frequency and fusion branches’ accurate results while avoiding or alleviating their significant errors through removing or averaging. Our cross-modality model improves accuracy by about 0.6 m and RMSE by about 1 m compared with the spatial branch.

### 4.3. Ablation Experiment

In this section, we conducted ablation experiments on our proposed cross-modality model in all datasets to verify the effectiveness of the bicubic interpolation and EMA module.

Table 5 shows the ablation experimental results. The experimental findings explicitly indicate that bicubic interpolation yields a 3% improvement in both positioning accuracy and RMSE. In contrast, the EMA module demonstrates a more pronounced enhancement, boosting these two metrics by approximately 13%. Notably, the combination of bicubic interpolation and the EMA module results in a 23% improvement in positioning accuracy and RMSE, which surpasses the performance achieved by either technique in isolation. Specifically, bicubic interpolation refines the features of each fingerprint via 2× super-resolution processing, thereby enhancing positioning accuracy and model robustness. Conversely, the EMA module significantly optimizes positioning performance by directing the neural network to focus on the valid regions of fingerprints. Based on these observations, we probably think that the EMA module substantially strengthens the model’s capability to learn details from input fingerprint images, while the richer details introduced by bicubic interpolation can be more precisely captured by the model. This mutually reinforcing effect ultimately leads to a marked improvement in positioning performance.

## 5. Conclusions

This paper proposes a novel fingerprinting positioning system using bicubic interpolation and a proposed cross-modality deep learning model. The bicubic interpolation is introduced to enhance the features of fingerprint images for more accurate and robust positioning by detailing the features of each fingerprint through super-resolution. Subsequently, the 2D FFT is performed on the enhanced fingerprint image to obtain the frequency domain fingerprint of each RP. Then, our cross-modality positioning model receives fingerprint spatial and frequency domain image inputs to give out the final 3D coordinates. Our proposed cross-modality model reduces the RMSE and average positioning errors through cross-modality fusion and multi-modal output combination, achieving more accurate and robust positioning. Experimental validation for our proposed cross-modality model was conducted on seven publicly available heterogeneous datasets. The results show that our proposed cross-modality model exhibits excellent performance and robustness compared with many traditional methods and one existing advanced time–frequency RSS fingerprinting method. In our future work, we plan to explore more effective strategies for fusing time–frequency data or its outputs. Additionally, we plan to combine different easily accessible data sources, such as Bluetooth and WiFi, in a lightweight manner to enhance the effectiveness and reliability of multi-modality positioning.

## Figures and Tables

**Table 1:** The fingerprint image size of all datasets used in this paper.

Dataset	Image Size (Before Interpolation)	Image Size (After Interpolation)
UJIB1 [30]	5 × 5	10 × 10
UJIB2 [30]	5 × 5	10 × 10
UEXB1 [31]	6 × 6	12 × 12
UEXB2 [31]	6 × 6	12 × 12
UEXB3 [31]	6 × 6	12 × 12
MINT1 [32]	4 × 4	8 × 8
ECS1 [33]	4 × 4	8 × 8

**Table 2:** Basic information for considered datasets.

Dataset	$N_{\mathrm{train}}$	$N_{\mathrm{test}}$	$N_{\mathrm{AP}}$	Area [$m^{2}$]	Technology
UJIB1 [30]	1680	420	24	151	BLE
UJIB2 [30]	2121	531	22	176	BLE
UEXB1 [31]	139	34	30	1000	BLE
UEXB2 [31]	184	46	30	1800	BLE
UEXB3 [31]	120	30	30	5800	BLE
MINT1 [32]	4973	810	11	1000	WIFI
ECS1 [33]	176,380	35,626	16	324	WIFI

**Table 3:** Performance evaluation results of mean 3D positioning error on all considered models and real-world datasets, with the best performing methods highlighted in bold.

Mean 3D Error (m)	Ours	[26]	CNN	DNN	1NN	W3NN
UJIB1 [30]	**3.61**	3.79	5.04	5.71	6.97	5.59
UJIB2 [30]	**1.91**	2.24	3.21	3.63	3.90	3.45
UEXB1 [31]	**3.84**	4.19	5.07	5.03	5.87	4.70
UEXB2 [31]	**4.13**	4.62	6.55	8.39	7.46	6.51
UEXB3 [31]	**6.82**	6.93	8.76	8.86	12.89	10.33
MINT1 [32]	**1.76**	1.95	3.22	3.67	3.73	3.65
ECS1 [33]	**1.92**	2.08	2.95	3.12	3.33	3.23
**Average**	**3.42**	3.69	4.97	5.49	6.31	6.18

**Table 4:** Performance evaluation results of RMSE on all considered models and real-world datasets, with the best performing methods highlighted in bold.

RMSE (m)	Ours	[26]	CNN	DNN	1NN	W3NN
UJIB1 [30]	**4.07**	5.00	5.65	6.44	7.99	6.23
UJIB2 [30]	**2.53**	2.71	3.52	3.91	4.67	3.89
UEXB1 [31]	**4.27**	4.95	5.91	5.93	7.33	5.81
UEXB2 [31]	**4.65**	5.43	8.49	10.72	9.92	8.74
UEXB3 [31]	**7.75**	8.11	10.39	10.26	17.00	13.11
MINT1 [32]	**2.15**	2.38	3.73	4.07	4.63	4.50
ECS1 [33]	**2.13**	2.51	3.38	3.77	4.07	3.99
**Average**	**3.92**	4.45	6.43	6.44	7.94	7.53

**Table 5:** Ablation experiment results for bicubic interpolation and EMA in our proposed cross-modality model.

Dataset	Bicubic	EMA	Mean 3D Error (m)	Gain	RMSE (m)	Gain
MINT1 [32]			2.66		3.39	
ECS1 [33]			2.49		2.81	
UEXB1 [31]			4.45		5.12	
UEXB2 [31]			5.65		6.57	
UEXB3 [31]			7.98		9.12	
UJIB1 [30]			4.42		4.96	
UJIB2 [30]			2.58		3.22	
**Average**			**4.32**		**5.03**	
MINT1 [32]	✓		2.57	3.4%	3.32	2.1%
ECS1 [33]	✓		2.41	3.2%	2.73	2.8%
UEXB1 [31]	✓		4.35	2.24%	5.09	0.59%
UEXB2 [31]	✓		5.21	7.80%	6.12	6.84%
UEXB3 [31]	✓		7.66	4.00%	8.79	3.60%
UJIB1 [30]	✓		4.35	1.60%	4.91	1.00%
UJIB2 [30]	✓		2.52	2.30%	3.15	2.17%
**Average**			**4.15**	**3.51%**	**4.87**	**2.73%**
MINT1 [32]		✓	2.02	24.10%	2.57	22.59%
ECS1 [33]		✓	2.17	12.85%	2.45	12.81%
UEXB1 [31]		✓	4.05	8.98%	4.65	9.11%
UEXB2 [31]		✓	4.86	13.98%	5.69	13.39%
UEXB3 [31]		✓	7.19	9.89%	8.23	9.75%
UJIB1 [30]		✓	3.97	10.18%	4.42	10.88%
UJIB2 [30]		✓	2.23	13.56%	2.89	10.25%
**Average**			**3.75**	**13.36%**	**4.42**	**12.68%**
MINT1 [32]	✓	✓	1.76	33.83%	2.15	36.6%
ECS1 [33]	✓	✓	1.92	22.90%	2.13	22.00%
UEXB1 [31]	✓	✓	3.84	13.70%	4.27	16.60%
UEXB2 [31]	✓	✓	4.13	26.90%	4.65	29.20%
UEXB3 [31]	✓	✓	6.82	14.50%	7.75	14.40%
UJIB1 [30]	✓	✓	3.61	18.30%	4.07	17.90%
UJIB2 [30]	✓	✓	1.91	30.00%	2.53	21.39%
**Average**			**3.42**	**22.88%**	**3.92**	**22.58%**

## Data Availability

No new data were created or analyzed in this study. Data sharing is not applicable to this article.
